# Comprehensive genomic and immunophenotypic analysis of CD4 T cell infiltrating human triple-negative breast cancer

**DOI:** 10.1007/s00262-020-02807-1

**Published:** 2020-12-10

**Authors:** He Zhang, Guohui Qin, Hui Yu, Xu Han, Sha Zhu

**Affiliations:** 1 Key Laboratory of Tumor Immunity, Center of Infection and Immunization, Department of Immunology, School of Basic Medical Sciences, Cancer Hospital Affiliated To Zhengzhou University, Zhengzhou University, Zhengzhou, 450001 People’s Republic of China; 2 Biotherapy Center, First Affiliated Hospital of Zhengzhou University, Zhengzhou, 450052 People’s Republic of China; 3 Henan Animal Health Supervision Institute, Zhengzhou, 450008 People’s Republic of China

**Keywords:** CD4^+^ T cell, Tils, Gene expression profile, Exhaustion, Chemotaxis, Degs

## Abstract

The aim of this study is to investigate the gene expression module of tumor-infiltrating CD4^+^T cells and its potential roles in modulating immune cell functions in triple-negative breast cancer. Differentially expressed genes were identified by comparison of the expression profile in CD4^+^T cells isolated from tumor tissues and peripheral blood of TNBC patients respectively. The differential expression analysis was conducted using R, and then the functional and pathway enrichment of the DEGs were analyzed using GSEA, followed by integrated regulatory network construction and genetic analysis of tumor-infiltrating immune cells based on a scientific deconvolution algorithm. As a result, abundant Treg and exhausted lymphocytes were detected, accompanied by largely decreased of effector/memory and cytotoxic T cells. Immune-related gene correlation analysis showed that the extent of follicular helper T cells gene expression signatures were inversely associated with those of CD4^+^ naive T cells and CD4^+^ memory resting T cells, but positively correlated with that of CD4^+^ memory activated T cells. In addition, we found five core genes including IFNG, CTLA4, FAS, CXCR6, and JUN were significantly over expressed in CD4^+^ TILs which may contribute to exhaustion of lymphocytes and participate in biological processes associated with regulation of chemotaxis. Study provides a comprehensive understanding of the roles of DEGs associated with the chemotactic and exhausted immunophenotypes of CD4^+^ TILs that are a valuable resource from which future investigation may be carried out to better understand the mechanisms that promote TNBC progression.

**Supplementary Information:**

The online version contains supplementary material available at 10.1007/s00262-020-02807-1.

## Introduction

Breast cancer is a prevalent malignancy in female globally, with ~ 1.4 million new cases diagnosed annually [1], of which triple‐negative breast cancer (TNBC) represents approximately 15–20% and have the highest genomic instability rates when compared with other breast cancer subtypes [2]. Previous studies have shown that TNBC can stimulate the immune system to form a tumor microenvironment (TME) where immune cells are the main component which can be identified as potential prognostic factors for TNBC patients [3]. Tumor-infiltrating lymphocytes (TILs) play an important role in promoting host protection against cancer and facilitating tumor escape from immune destruction [4, 5]. The intensity of tumoral immune response influences the effectiveness of cancer therapy and is also correlated with favorable clinical outcome of the patients [6].

Most neoantigens of cancer cells can stimulate T cells and also induce a regulatory immune response [7, 8]. Intratumoral CD4^+^ T cells are increasingly recognized as responsible for antitumor immune response and the production of inflammatory mediators that induce tumor growth, invasion, angiogenesis, and metastasis which are associated with clinical outcomes [9, 10]. Study reported that increasing numbers of CD4^+^ T cells correlated significantly with improved disease-specific survival and that a high density of stromal CD4^+^ T cells was a favorable independent prognostic factor in non-small cell lung cancer (NSCLC) patients [11]. Accumulating evidence confirmed the prognostic value of CD4^+^ T cells for many other tumors [12–15] and it has been documented that high intensity of intratumoral CD4^+^ T cell is associated with a better outcomes [15, 16].

The expression profiles of the intratumoral CD4^+^ T cells can be used to analyze tumor-infiltrating T cell subsets and to predict how a patient will respond to cancer therapy [17]. A comprehensive understanding of the intratumoral T cell characteristics contributes to early diagnosis and precise treatment [18]. Study has shown that an increased proportion of TILs, such as CD4^+^ T and CD8^+^ T cells, was associated with better clinical outcomes in breast cancer [19]. It has been proposed that the characterization of TILs, like their immunophenotype, could help screen immunotherapy clinical trials [20]. Numerous studies have demonstrated that tumor immunotherapy, in particular, activation of intratumoral helper T cells, is an alternative or complement to chemotherapy or surgery [21–23]. Study indicated that blockade of the immune checkpoint protein cytotoxic T-lymphocyte-associated protein 4 (CTLA4) enhance the antitumor immune responses ability of the immune cells in TME [24]. With the effective clinical usage of immune-targeted therapies, a key issue is to identify the biological and genetic factors of intratumoral lymphocytes involved in the immune response.

On that basis, the aim of this study was to investigate the gene expression profile of tumor-infiltrating CD4^+^ T cells and to predict their potential roles in modulating antitumor immune function. In this work, we use microarray data to analyze the phenotype and gene expression characteristics of tumor-infiltrating CD4^+^ T cells from TNBC patients. This study might provide clues for better understanding the phenotype of these cells in particular attenuation of immune suppression which may offer clinical benefit for controlling and suppressing tumor progression.

## Subjects and methods

### Patients and samples

All human samples used in the experiments were tested in accordance with the ethical principles of Zhengzhou University. Patients with TNBC who had not received chemotherapy or radiotherapy were included in the present study such as microarray, quantitative real-time PCR, immunohistochemical, and immunofluorescence evaluation (Table 1).

**Table 1:** Demographics and characteristics of the study group (n = 61)

Variable	Number or characteristics
Mean age at diagnosis	54.3
Median age at diagnosis	55
Standard deviation	9.14
T-stage	
II	35
III	36
N-stage	Positive
ER status	Negative
PR status	Negative
HER2 status	Negative
Neoadjuvant chemotherapy	Not received

CD4^+^ T cells isolated from primary tumor tissues were compared by microarray and RT-qPCR with the cells from peripheral blood (PB) of the TNBC patients respectively. All patients agreed with their surgical or biopsy specimens to be used in research. CD4^+^ T cells were positively purified by microbeads (Miltenyi Biotec, Germany) and AutoMACS (Miltenyi Biotec). All the processes were carried out according to the manufacturers’ instructions. Flow cytometry was used to control the cell purification with the purity of negatively samples > 98%, and the purity of positive TILs samples was > 95%.

### Microarrays and DEG identification

The isolated CD4^+^ T cells from tumors and PB were used for affymetrix microarray. Total RNA from CD4^+^ T cells used for microarray was extracted using an RNeasy Mini kit (Qiagen, Valencia, CA). Amounts, purity, and integrity of RNA were assessed on a spectrophotometer (NanoDrop, Nyxor Biotech). High-quality total RNA was labeled following the manufacturer’s protocols for probe preparation and hybridization on the Affymetrix U133 Plus 2.0 GeneChip. Affypackage in R was used to normalize and convert CEL files to expression data [25, 26]. The differentially expressed genes (DEGs) were subsequently calculated using the Limma package, based on the false discovery rate controlling procedures [27]. |logFC|> 1 and *P* < 0.05 were used as comparison criteria and threshold to define the differentially expressed genes for CD4^+^ T cells between TNBC and PB samples.

### Functional and pathway enrichment analysis

Gene Ontology (GO) enrichment analysis was performed using Database for Annotation, Visualization and Integrated Discovery (DAVID) [28]. The DEGs in the tumor-infiltrating CD4^+^ T cells of breast cancer patients were screened for functional enrichment. The potential functions of the DEGs in biological processes, molecular functions and cellular components were predicted by GO analysis. In addition, systematic analysis of differences in gene functions was conducted using the Kyoto Encyclopedia of Genes and Genomes (KEGG) database [29]. GO functional and KEGG pathway enrichment analyses were performed for the up-regulated and down-regulated genes, respectively. The terms with *P*-value < 0.05 and count ≥ 2 were considered the cut-off criterion.

### GSEA analysis of the DEGs

Gene Set Enrichment Analysis (GSEA) analysis [30] based on predefined gene sets from the Molecular Signatures Database (MSigDB v5.0, http://software.broadinstitute.org/gsea/msigdb/index.jsp) was used to assesses whether an a priori defined set of genes shows statistically significant, concordant differences between two biological states. A gene set is a group of genes that shares pathways, functions, chromosomal localization, or other features. For the present study, we used “C7. all. V7.2 symbols. gmt” collection sets for GSEA analysis and list of ranked genes based on a score calculated as − log10 of *P*-value multiplied by the sign of fold-change. The number of permutation is set to 1000.

### Evaluation of TILs

The analytical tool CIBERSORT (Cell-type Identification By Estimating Relative Subsets Of RNA Transcripts) was used to determine relative proportions of immune cells through deconvolute mixed samples [17]. The CIBERSORT is a deconvolution algorithm, based on 22 immune cell reference profiles, which uses a “signature matrix” of genes’ expression values to characterize immune cell composition [31]. In other words, the 22 cell subpopulations of the tumor microenvironment were evaluated using the CIBERSORT to analyze the normalized gene expression values. The immune subpopulations from TILs and PB were assessed employing two different unsupervised approaches: the hierarchical clustering method and PCA (R-bioconductor, *stats*package) which performed using the *ComplexHeatmap* package within R-bioconductor. The samples with *P* < 0.05 were included.

### PPI network and module analyses

STRING (version 10.0, http://www.stringdb.org/) is an online database retrieving gene interactions to provide experimental and predictive PPI (protein–protein interaction) information [32]. Cytoscape software (version 3.2.0, http://www.cytoscape.org) was used to construct PPI networks [33] to visualize the interaction of the up-regulated and down-regulated genes [34]. Subsequently, the degree centrality of the nodes was calculated by the CytoHubba plug-in [35] software. The hub proteins were identified with higher degrees of centrality [36]. Additionally, the KEGG pathway enrichment analysis for nodes in the significant modules was performed using MATHT tool.

### Gene validation using RT- qPCR

We selected patients who were confirmed diagnostic of triple-negative breast cancer. Total RNA from CD4^+^ T cells was extracted by Trizol reagent (Takara, Dalian, China). Conversion of the mRNA to cDNA was performed using a Taqman Reverse Transcription kit (Roche, Branchburg, NJ) according to the manufacturer’s instructions. Real-time quantitative PCR was performed using QPK-201 SYBR Green master mix (Toyobo, Osaka, Japan) and the ABI 7300 system from Applied Biosystems. The thermocycling protocol was set as an RT step at 50 °C for 20 min, DNA polymerase activation step at 95 °C for 2 min and total 35 PCR cycles (95 °C for 20 s, 60 °C for 30 s) [29]. The primers used in this study were synthesized from Invitrogen (Beijing China). All reactions were performed in triplicate. Comparative CT method was used to calculate the fold change of expression in each gene. Expression data are described by a log-ratio calculated by comparing ΔCq from the tumor-infiltrating CD4^+^ T cells with ΔCq from the controls.

### Algorithmic evaluation of the immune cells by ImmuCellAI

Immune Cell Abundance Identifier (ImmuCellAI), a gene set signature‐based method, was designed to estimate the abundance of the immune subsets from gene expression data. For maintaining the correlative structure of real data and controlling the mixing ratios of immune cell components, the gene–gene covariance matrix was calculated for all genes in using gene expression data. Subsequently, immune cell subsets were randomly sampled from uniform and length *n* was calculated, which was the average of gene expression in the reference profiles of the immune cell types. Next, a vector of length *n* was sampled from the multivariate normal distribution with mean length and covariance. For screening marker genes, the average correlation between gene expression data in tumor-infiltrating CD4^+^ T cells from TNBC patients and control samples was calculated using the Pearson correlation for all samples. Next, for each marker gene per immune cell, the standard correlation deviation among the cell with other cells was calculated, and genes with standard deviation larger than 1.5 were selected.

### Immunohistochemical and immunofluorescence evaluation

Immunohistochemistry (IHC) was conducted using sections (4 μm) of formalin-fixed, paraffin-embedded tumor tissues from patients with triple-negative breast cancer. Following deparaffinization and rehydration of the tissue sections, antigen retrieval was performed by microwaving in 10 mM citrate buffer (pH 6.0). After blockade of endogenous peroxidase, mouse anti-CTLA4, CXCR6, and FAS primary monoclonal antibodies (Agilent Technologies) and peroxidase-conjugated, rabbit anti-mouse secondary antibody (Agilent Technologies) were sequentially applied at 1:50 and 1:500 dilution respectively. The sections were visualized by diaminobenzidine (DAB) and counterstained with hematoxylin. For immunofluorescence staining, after deparaffinizing, rehydrating, antigen retrieval and endogenous peroxidase activity blocking, Cy3 or FITC (BioLegend, USA) was used as a secondary antibody (1.5 μg/mL) for one hour, and nuclei were counterstained by 4′-6-diamidino-2-phenylindole (DAPI; Sigma, USA) for 10 min [37]. In TUNEL assay, TDT enzyme, dUTP and buffer from the TUNEL kit were mixed at a ratio of 1:5:50. The reaction solution was added on the tissue placed in a flat wet box. Finally, after dehydration and mounting, the tissue sections were observed under a microscope (Olympus BX51, JP) at a magnification of 200× . At least six sections of one tumor tissue were used for quantitative evaluation.

### Statistics

Two-tailed Student’s *t* test with unequal variance (or a *χ*^2^ test) was used to calculate the *P* values for the experimental data. *P* < 0.05 was considered significant. Data are shown as the mean ± SD.

## Results

### Gene Expression Profile of CD4^+^ TILs in TNBC

**Fig. 1:**
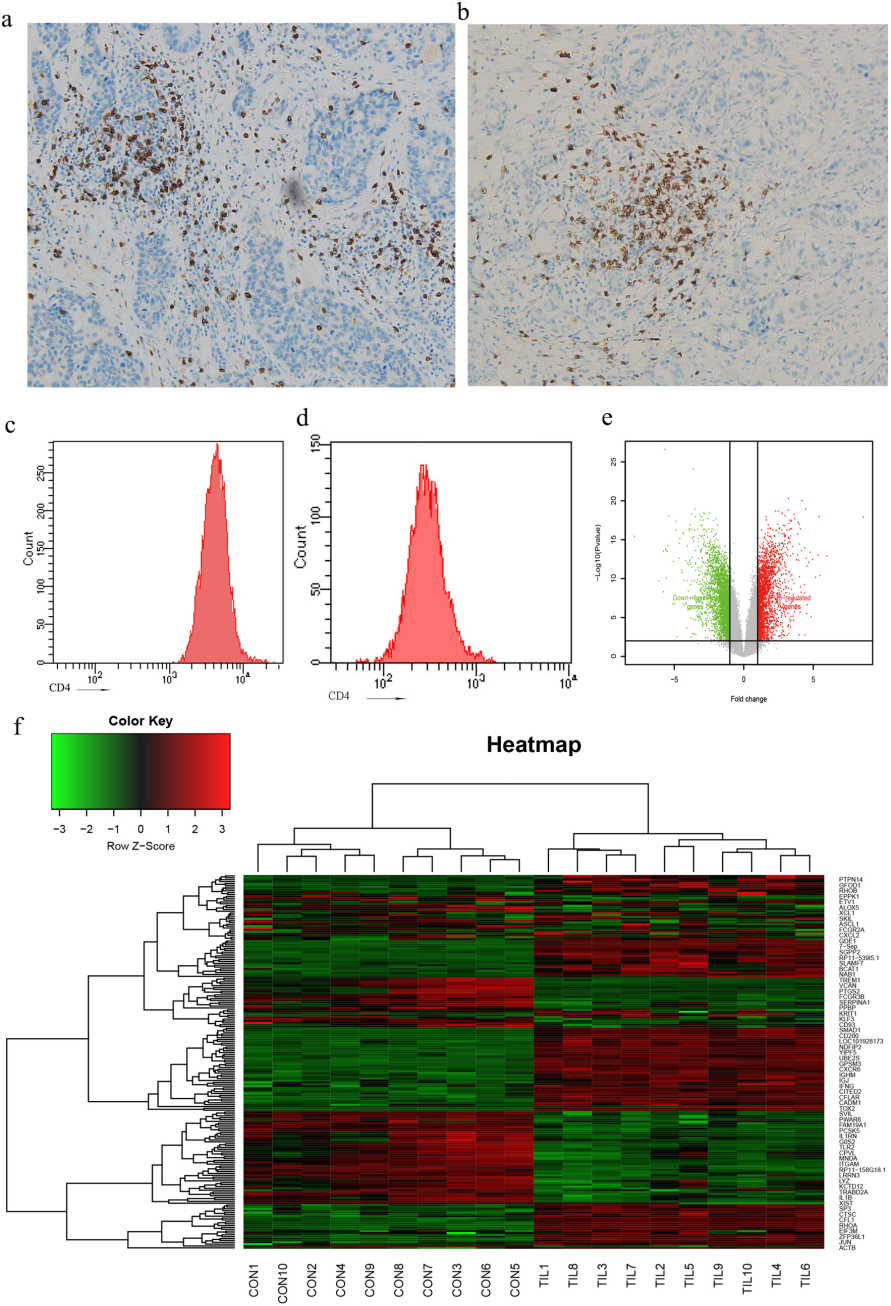
Microarray analysis for screening the DEGs in isolated CD4^+^ T cells. Detection of CD4^+^ T cells by immunohistochemistry in representative TNBC samples **a** CD4^+^ T cells in stroma, **b** CD4^+^ T cells in stroma and infiltrated among the cancer cells. Flow cytometric analysis was performed immediately after CD4 ^+^ T cells isolated from PB **c** or TNBC tissues **d**. **e** Volcano plots of DEGs from analyzed microarray data. **f** The dendrogram of DEGs in the CD4^+^ T cells. Red represents higher expression and green lower expression. The criteria used to select DEGs were *P* < 0.05 and |logFC|> 1. DEGs, differentially expressed genes

The distribution of CD4^+^ TILs were detected by IHC in samples of TNBC and normal breast tissue, as shown in Fig. 1a, b and Supplementary Fig. 1a a large number of CD4^+^ T cells were frequently distributed in stromal compartments within tumor borders, while some of the cells scattered within tumors area. In addition, The proportion of CD4^+^ T cells in normal and tumor tissue were quantitatively analyzed and showed in Supplementary Fig. 1b–d. We purified CD4^+^ TILs from 10 TNBC patients who were previously treatment-naive for microarray. After purification, mean purities for CD4^+^ T cells from tumors and PB were 99.4% (98.3–99.7%) and 99.1% (97.6–99.5%) respectively (Fig. 1c, d). To identify differentially expressed genes (DEGs) between CD4^+^ T cells from TNBC and from PB, threshold |logFC|> 1 and *P* < 0.05 was used as criteria for comparison. Results showed that a total of 2968 DEGs were identified and among them, 1431 were down-regulated and 1537 were up-regulated (Fig. 1e). The dendrogram of top DEGs in CD4 ^+^ T cells was shown in Fig. 1f.

### Functional annotation and pathway enrichment of DEGs

**Fig. 2:**
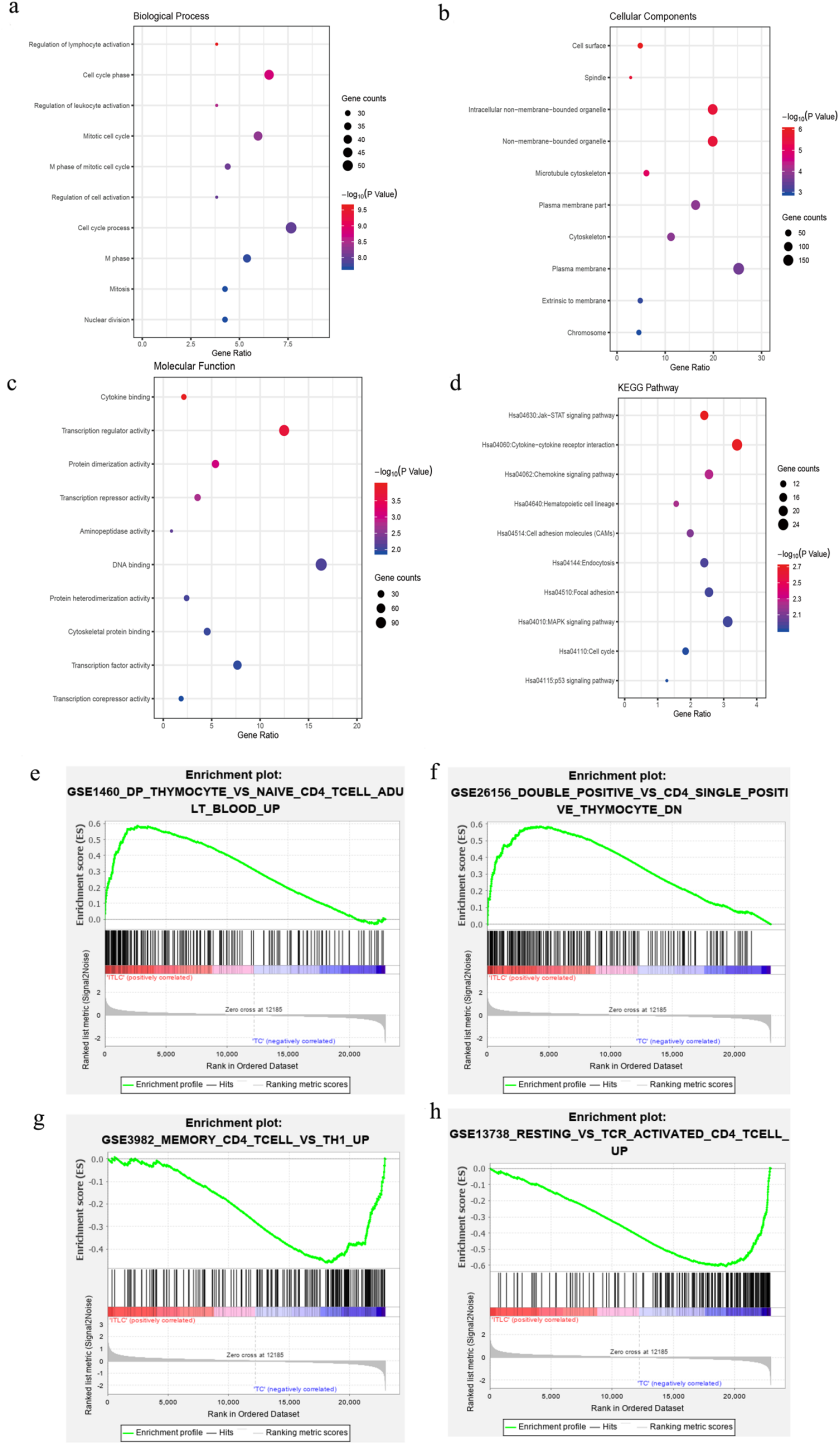
GO, KEGG and GSEA analyses of DEGs. **a–c** GO analyses. Shown are the top 10 biological processes **a**, cellular components **b**, and molecular functions **c**. **d** KEGG pathway analysis. **e** Enrichment of genes in DP_THYMOCYTE_VS_NAIVE_CD4_T CELL_ADULT_BLOOD_UP by GSEA. **f** Enrichment of genes in DOUBLE_POSITIVE_VS_ CD4_SINGLE_ POSITIVE_THYMOCYTE_DN by GSEA. **g** Enrichment of genes in MEMORY _CD4_TCELL_VS_TH1_UP by GSEA. **h** Enrichment of genes in RESTING_VS_ TCR_ACTIVATED_CD4_TCELL_UP by GSEA. The GSEA software was used to calculate the enrichment levels

Gene Ontology (GO) and Kyoto Encyclopedia of Genes and Genomes (KEGG) pathway analysis were performed to explore the unique role of candidate genes. The top ten biological process terms were mainly involved in the regulation of lymphocyte and leukocyte activation, cell cycle phase, and mitotic cell cycle (Fig. 2a). With regard to the cellular components, the DEGs were primarily associated with cell surface, spindle, and intracellular non-membrane-bounded organelle (Fig. 2b). In terms of molecular function, it indicated that the DEGs were mostly enriched in cytokine binding, transcription regulator activity, and protein dimerization activity (Fig. 2c). Followed KEGG pathway enrichment analysis showed that the DEGs were basically enriched in Jak − STAT signaling pathway, cytokine − cytokine receptor interaction, and chemokine signaling pathway (Fig. 2d). The top 5 enriched terms are presented in Table 2.

**Table 2:** GO functional and KEGG pathway enrichment analysis of DEGs

ID	Terms	Count	*P* value	Genes
*Biological process*				
GO:0051249	Regulation of lymphocyte activation	27	2.37E−10	LST1, VCAM1, STAT6, CD47, IFNG, ZAP70, BCL6, FAS, IL6, CTLA4…
GO:0022403	Cell cycle phase	46	1.62E−09	BCAT1, NEK2, TTK, BCL2, EGFR, CDK1, KIF15, PIM1, TPX2, ATM…
GO:0002694	Regulation of leukocyte activation	27	3.09E−09	CD74, VCAM1, STAT6, CD47, IFNG, ZAP70, BCL6, FAS, CD24, IL6, CTLA4, LAT, CD38, CD80…
GO:0000278	Mitotic cell cycle	42	5.05E−09	BCAT1, HAUS5, NEK2, TTK, BCL2, EGFR, CDK1, KIF11, KIF15, PIM1…
GO:0000087	M phase of mitotic cell cycle	31	8.52E−09	HAUS5, NEK2, NEDD9, CEP55, PTTG1, CCNG2, TUBB, NCAPG, BUB1, BUB3…
*Cellular components*				
GO:0009986	Cell surface	34	9.58E−07	CAV1, CXCR2, TIMP2, SDC4, CD74, VCAM1, FAS, CD6, IL2RB, IL6R…
GO:0005819	Spindle	20	2.49E−06	CDK1, HAUS5, KIF11, NEK2, DLGAP5, KIF15, TPX2, TTK, MID1, ATM…
GO:0043232	Intracellular non-membrane-bounded organelle	140	2.64E−06	STRN, TTK, PDLIM1, MRPS31, KLHL3, KIF13A, ANK3, ASPM, OXR1, GNL3…
GO:0043228	Non-membrane-bounded organelle	140	2.64E−06	VAPA, PDLIM5, STRN, TTK, KLHL3, HOOK1, KIF13A, ANK3, OXR1, GNL3…
GO:0015630	Microtubule cytoskeleton	43	9.16E−06	HAUS5, VAPA, NEK2, TTK, NEDD9, EZR, BUB1, CDK1, KIF11, NIN…
*Molecular functions*				
GO:0019955	Cytokine binding	15	1.01E−04	IL2RB, NOG, TGFBR2, IL6R, CXCR3, IL11RA, CCR9, CCR5, CXCR6, CXCR5…
GO:0030528	Transcription regulator activity	88	2.00E−04	FOSL2, BACH2, PTTG1, EGR1, CTBP2, YY1, SCAI, LEF1, FOSB, ID3…
GO:0046983	Protein dimerization activity	38	9.01E−04	NOG, FOSL2, BACH2, TPD52, BATF, BCL2, FOSB, IL6R, FOXP1, JUN…
GO:0016564	Transcription repressor activity	25	0.001789	HOXC6, AES, BCL11A, BCL6, SKIL, TCF4, BCOR, IKZF4, SCAI, FOXP1…
GO:0004177	Aminopeptidase activity	6	0.006034	LNPEP, ERAP1, DPP8, ERAP2, PHEX, DPP4
*KEGG pathways*				
hsa04630	Jak-STAT signaling pathway	17	1.95E−03	IL2RB, IL6, STAT5B, PIM1, IL6R, SOCS5, IL11RA, STAT6, IFNG, AKT3…
hsa04060	Cytokine-cytokine receptor interaction	24	2.03E−03	EGFR, IL2RB, IL6, TNFSF4, CXCR2, IL6R, CXCR3, IL11RA, IL17RA, FAS…
hsa04062	Chemokine signaling pathway	18	5.44E−03	VAV3, STAT5B, CXCR2, CXCR3, PRKCB, CCR9, CCR5, RAC2, CXCL13, AKT3…
hsa04640	Hematopoietic cell lineage	11	5.90E−03	CD38, CR1, GP5, IL6, CD37, TFRC, CD59, CSF3R, IL6R, ITGA4…
hsa04514	Cell adhesion molecules (CAMs)	14	7.55E−03	CADM1, CTLA4, CDH1, ITGA4, SDC4, VCAM1, CD80, CD58, HLA-DOA, CD6…

It is showed that the differentially expressed genes are mainly involved in lymphocytes activation and mitosis the in biological process (Table 2). Results revealed the proteins of IFNG, CXCR6, and CD80 shared the same critical pathways like regulation of lymphocyte adhesion, whereas CTLA4 and FAS participated in negative regulation of lymphocyte exhaustion and programmed cell death respectively. Moreover, Gene Set Enrichment Analysis (GSEA) was implemented between TNBC and PB groups. Results revealed that gene sets were largely enriched in double-positive lymphocytes and activated CD4^+^ T cells (Fig. 2e–h). High expression levels of FAS and CTLA4 were found in CD4^+^ T cells from TNBC samples. Increasing evidences support that CTLA4 was highly expressed in Treg with essential roles in repressing anticancer immunity and maintaining self-tolerance [38]. FAS molecules in tumor-infiltrating lymphocytes interacting with FAS ligand may result in the increase of apoptosis of these cells. These analyses reflect an activated or exhausted state of the CD4^+^ the TILs.

### Visualization and evaluation of CD4^+^ T-cell subsets infiltration

**Fig. 3:**
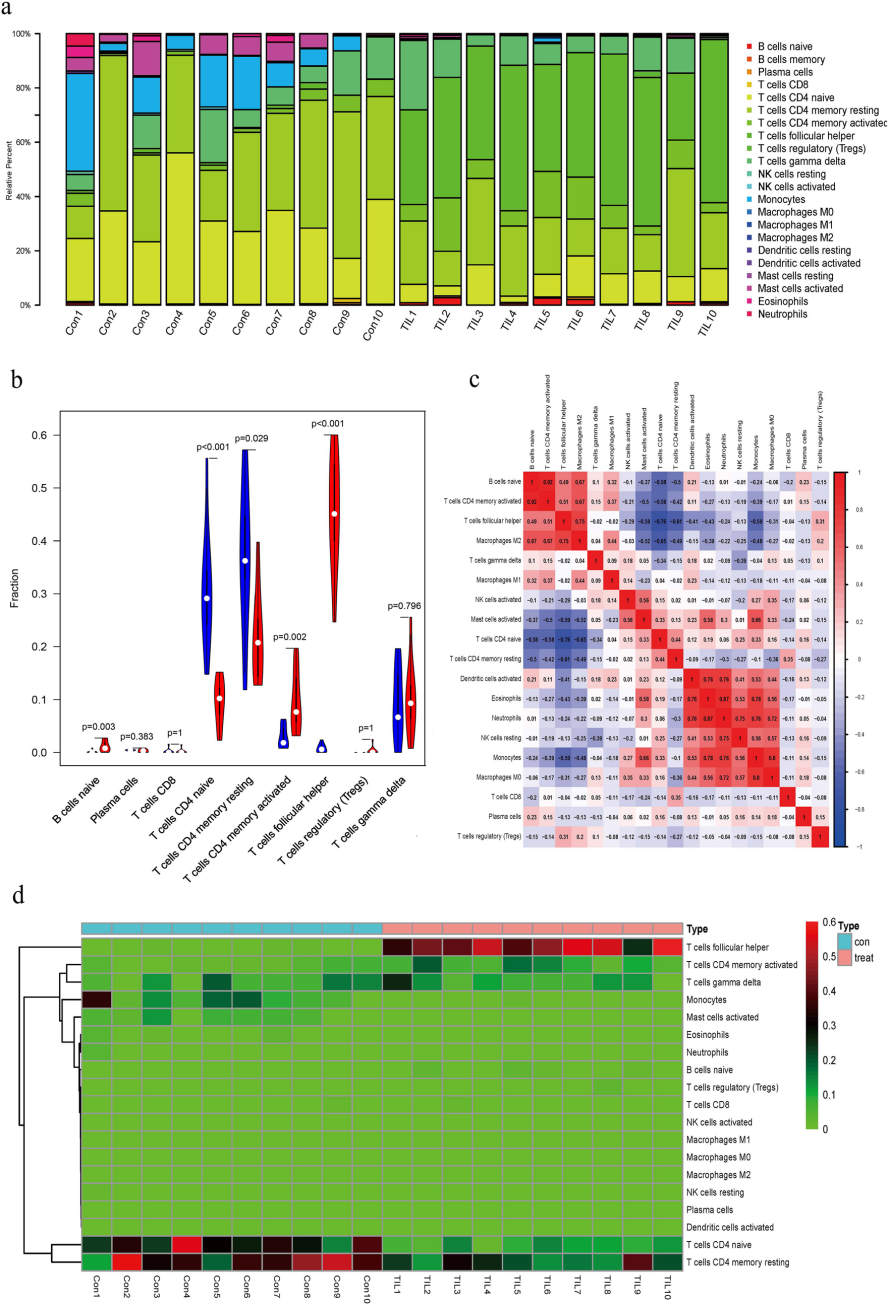
Landscape of CD4^+^ T-cell subsets infiltrated in TNBC. **a** Bar plot of immune infiltration of the isolated CD4^+^ T cells from TNBC tissues and PB samples. **b** The violin plot of the immune cell proportions. **c** Correlation matrix of all the immune cell proportions between TNBC tissues and PB samples. **d** Heat map of the 22 immune cell proportions between TNBC tissues and PB samples. Pearson chi-squared test was utilized to perform correlation analysis

CIBERSORT, a deconvolution algorithm method based on gene expression, was used to predict the constituent of multiple immune cell types in the tumor tissue admixtures. The cellular composition can be evaluated based on standardized gene expression value, which indicates the abundances of specific cell types. In this study, we first explored CD4^+^ TILs in TNBC tissue by the CIBERSORT algorithm. Various proportions of the CD4^+^ T-cell subsets in each TNBC sample were shown in different colors, and the levels of the immune cell populations were displayed by the length of the bars in the bar chart (Fig. 3a). From the chart of Fig. 3b, we identified a relatively high percentage of follicular helper T cells (*P* < 0.001), whereas a significantly low percentage of CD4^+^ naive T cells was found in the TILs (P < 0.001). Result revealed that T cells follicular helper were inversely correlated with T cells CD4^+^ naive and memory resting T cells (*r* = − 0.76 and *r* = − 0.61, respectively), which suggested an antagonistic function of Tfh with the two cell types in TNBC (Fig. 3c). The correlations between these differentially expressed types of immune cells were shown in Fig. 3d. Based on the 22 immune cells subpopulation, the isolated TILs were further divided into 2 discrete groups using the hierarchical clustering. The aberrant immune cell infiltration especially the increased CD4^+^ memory activated T cells may have an important clinical value in TNBC.

### Identification of hub genes and analyzing the modules of the networks

**Fig. 4:**
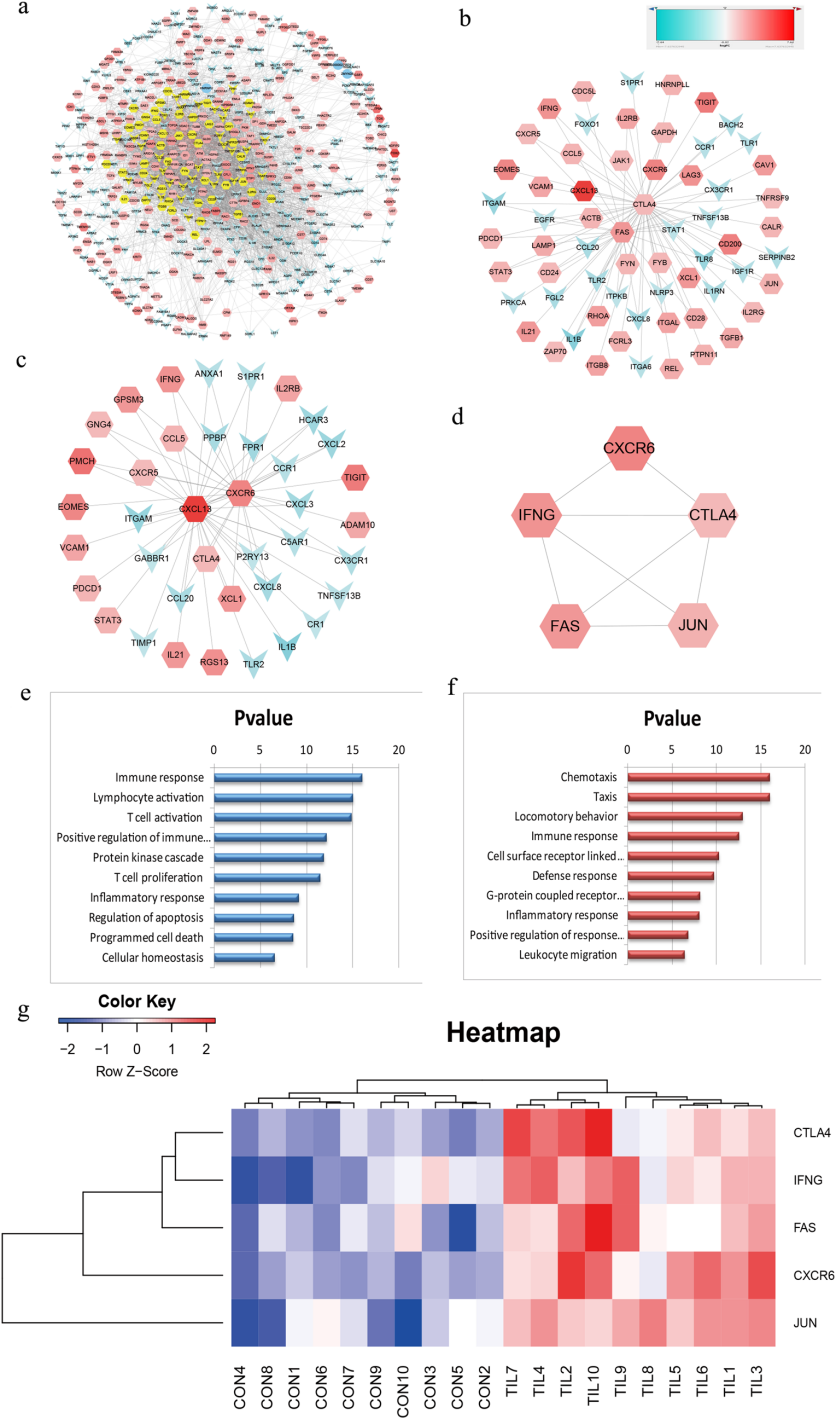
PPI networks and involved signal pathways. **a** Network of the DEGs from the microarray data. **b** Network derived from panel A with first neighbors associated with the core proteins CTLA4 and FAS. **c** Network derived from panel A with first neighbors associated with the core proteins CXCL13 and CXCR6. **d** Significant hub nodes extracted from network **b**. **e** Signal pathways involved in network **b**. **f** Signal pathways involved in network **c**. **g** Heatmap of the significant hub genes

To investigate the gene expression module of tumor-infiltrating CD4^+^ T cells results in the influence of tumor microenvironment, protein–protein interaction (PPI) networks were generated using the significantly expressed DEGs (Fig. 4a). To characterize the attributes of the key nodes in the PPI network, two networks mainly associated with the core proteins were identified to be candidate markers, which may exert significant influence on the biological function of the CD4^+^ T cells (Fig. 4b, c). The two networks consisted of 63 nodes, 87 edges and 39 nodes, 61 edges respectively. To further identify the hub genes and key pathways, EPC (Edge Percolated Component) and shortest path analyses were conducted by CytoHubba plug-in in Cytoscape. It is worth noting that CXCR5 was differentially up-regulated and directly interacted with CXCR6 (Fig. 4c). The most significant module composed of five nodes including FAS, IFNG, CXCR6, CTLA4 and JUN were screened out from the network with a connectivity degree > 18 (Fig. 4d). Functional annotation and pathway enrichment of these nodes in the above-mentioned networks showed that they were primarily associated with activation of immune response and chemotaxis (Fig. 4e, f). The heatmap demonstrated that all of the five hub genes are significantly up-regulated in the TILs of the TNBC samples (Fig. 4g). Furthermore, the expression pattern of CD4^+^ T cells isolated from peripheral blood of TNBC patients was compared with that of healthy donor blood. Protein–protein interaction (PPI) network was generated using the significantly expressed DEGs (Supplementary Fig. 2a). Two major networks related to the core nodes were presented in Supplementary Fig. 2b, c in which four significantly up-regulated genes including CTLA4, JUN, CXCR6, and CXCL13 whose expression levels also markedly increased in CD4^+^ T cells infiltrated in TNBC tissue compared with the cells in peripheral blood of TNBC patients. However, FAS was not found highly expressed in the tumor “macro”-environment. Moreover, CXCL13, IFNG, FN1, SPP1, and CXCR6 were screened as the hub genes from the significantly expressed DEGs and showed in Supplementary Fig. 2d.

### Validation of the chemotaxis and immune response-related hub genes

**Fig. 5:**
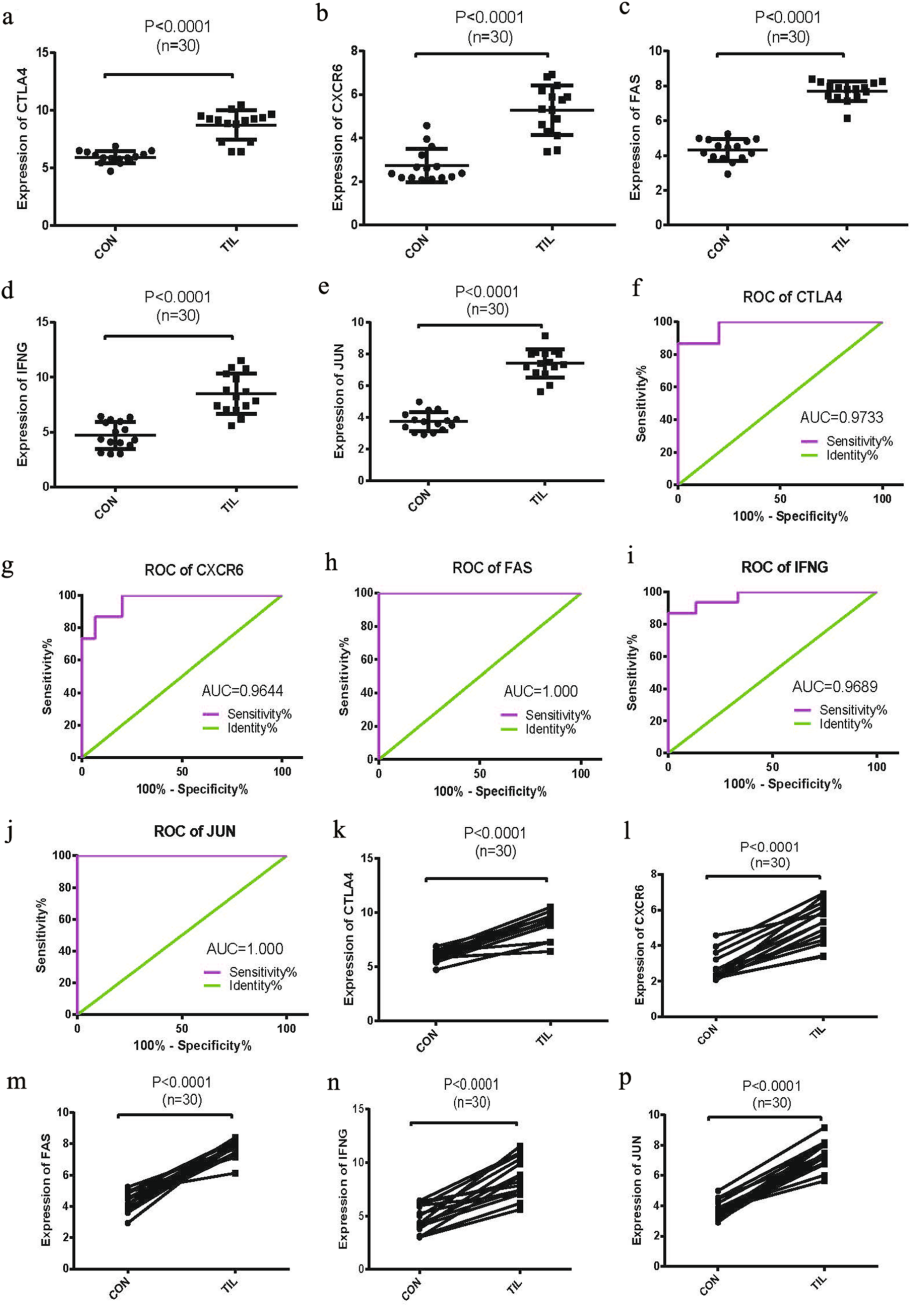
Comparable evaluation of the expression patterns and ROC analysis of the five differentially expressed genes. **a**–**e** Expression levels of CTLA4, CXCR6, FAS, IFNG, and JUN in CD4^+^ T cells isolated from TNBC tissues and PB samples respectively. **f**–**g** ROC curves to estimate the consistency of the expression values between TNBC tissues and PB samples. **k**–**p** Expression linear correlations between the two groups of TNBC and PB samples

The expression pattern of the above five differentially expressed hub genes in clinical samples were evaluated by quantitative PCR and shown as Fig. 5a–e. Results showed that mRNA expression levels of these genes were significantly increased in tumor-infiltrating CD4^+^ T cells compared with the cells from the PB of TNBC patients. ROC (receiver operating characteristic) analysis was then analyzed for all of the hub genes (Fig. 5f–j), and areas under the curve (AUC) of CTLA4, CXCR6, FAS, IFNG, and JUN were 0.9733, 0.9644, 1.000, 0.9689 and 1.000, respectively (*P* < 0.01). The AUC represented the set of all possible statistical tests of the expression data with equal probability for a true positive and a false positive result based on each decision threshold value [39]. Expression linear correlations between two groups among these hub genes were shown as Fig. 5k–p, in which CTLA4, FAS and JUN were in good consistency.

### Significance of extensive infiltration of CD4^+^ TIL subsets and key proteins identification

**Fig. 6:**
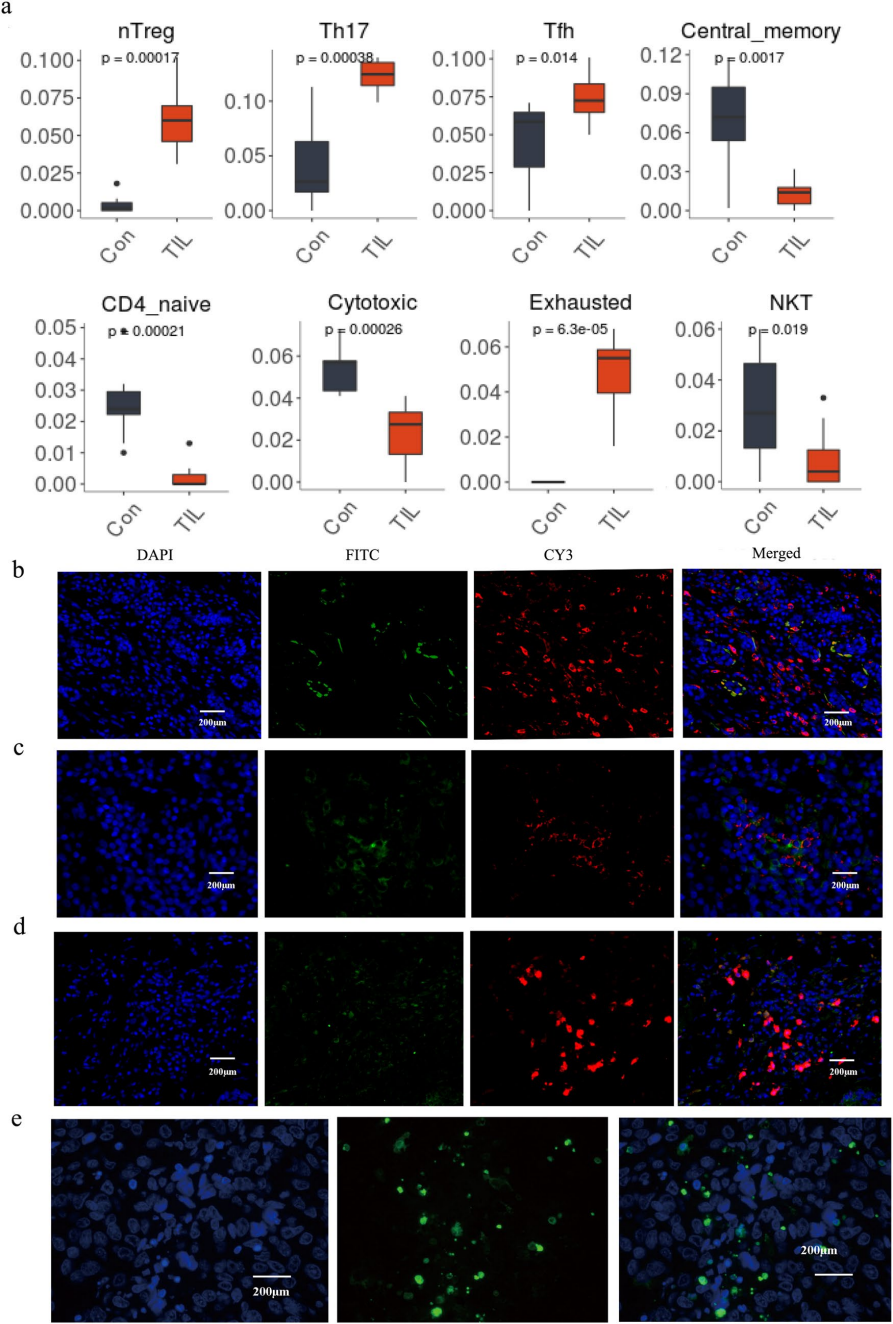
CD4^+^ T-cell subset profiles and immunofluorescence image of CTLA4, CXCR6, FAS in TNBC tissue. **a** CD4^+^ T cell subset profiles in the TNBC. Immunofluorescence labeling for **b** CD4 (red) and CTLA4 (green), **c** CD4 (red) and CXCR6 (green), **d** CD4 (red) and FAS (green) as well as merged images. **e** Detection of the apoptosis cells infiltrated in the tumor tissues. (*n* = 6, *P* < 0.05, 200X)

The presence of CD4^+^ T cell subset profiles in the TILs was reanalyzed applying IMMUCELL AI criteria. Results showed that the intratumoral CD4^+^ T cell profiles were significantly enriched in nTreg (*P* < 0.001), Th17 (*P* < 0.001), Tfh (*P* = 0.014), and exhausted cells (*P* < 0.001) whereas the central memory (*P* = 0.0017), CD4 naive (*P* < 0.001), cytotoxic (*P* < 0.001), and NKT cells (*P* = 0.019) were significantly decreased in the CD4^+^ TILs (Fig. 6a), which is consistent with the results analyzed by CIBERSORT algorithm. Immunofluorescence labeling of CD4^+^ T cells infiltrated in normal breast tissue was performed (Supplementary Fig. 3a) and the proportion of the CD4^+^ T cells were evaluated and showed in Supplementary Fig. 3b–e. To further illustrate the immune cell profiles, we detected the apoptotic cells by TUNEL staining assay and observed that the apoptotic TILs are significantly increased in the TNBC microenvironment (*P* < 0.001). We also found the increased expression of the protein of CTLA4, CXCR6, and FAS in the CD4^+^ TILs of the clinical samples (Fig. 6b–e). In addition, apoptotic cells infiltrated in the normal breast tissues were detected (Supplementary Fig. 3g) and quantitative evaluation of the apoptotic T cells in breast tumor and normal tissue were presented in Supplementary Fig. 3f, h.

## Discussion

Tumor-infiltrating lymphocytes are a kind of mononuclear immune cells. The intratumoral lymphocytes phenotypes consisted of varying proportions of CD4^+^ T cells, CD8^+^ T cells, B cells and NK cells, whereas, T cells were the main phenotype and significantly associated with pathological characteristics of the patients. A growing number of studies have suggested that TILs can be used to predict clinical outcome and treatment response of various tumors [40, 41]. It is indicated that the diversified CD4^+^ T cell cloning responses may reflect the diversification of neoantigens of cancer cells. Actually, studies showed that increased infiltration of memory T cells and mature T cells are closely associated with a favorable prognosis [42], but immunosuppressive regulatory T cells are opposite [43].

In this study, it is showed that infiltrated lymphocytes were significantly increased in the TME of triple-negative breast cancers (TNBCs). Furthermore, the relationship between the Th gene expression profile and the level of lymphocytes was investigated, which reflects the degree of TILs infiltration in TNBC samples. Another study also demonstrated increased infiltration of TILs in TNBC tissues [44] which is consistent with our results. In addition, our analytic result showed that cytotoxic and central memory T cells were significantly reduced, whereas regulatory T cells, Tfh and exhausted T cells were significantly increased in TNBC patients (Fig. 6a). Traditionally, activated memory T cell-mediated immunity is considered to be antitumoral, since patients with higher levels of active memory T cells are predicted to have longer-term disease-free survival. However, tumors infiltrated with polarized Treg and Tfh subpopulations were suggested an immunosuppressive microenvironment and a poorer prognosis of TNBC patients [45]. Th17 cells have been shown to work synergistically with IFN-γ to produce important antitumor chemokines CXCL10 and CXCL9; however, research indicated that IL-17 was a predictor of poor prognosis in hepatocellular carcinoma patients [46], suggesting the antitumor and protumor immune responses reports may be context dependent.

From the network, we found that the expression levels of FAS and CTLA4 were markedly increased in intratumoral CD4^+^ T cell of TNBC samples. As a death receptor, FAS recruits the adaptor protein procaspase-8 when it interacts with FasL to form a death‐inducing signal complex (DISC), consequently leading to the proteolytic stimulation of apoptosis [47]. Previous studies have shown that IFN-γ treatment could up-regulate Fas expression level in multiple cell types and tumors as well as promote Fas-mediated apoptosis [48, 49]. CTLA-4, as an inhibitory immune checkpoint, the expression level of which was remarkably higher in basal-like TNBC and HER2-enriched tissues compared with the other BC molecular subtypes [50]. CTLA4 plays an important role in negatively immunomodulating the activity of T lymphocytes via cell extrinsic and intrinsic pathways. Cell extrinsic events such as the competition with CD28 in binding to its legends CD80/86, lead to T cell unresponsiveness due to inhibitory signals delivered [51]. Intrinsic events include the inhibition of protein translation, activation of ubiquitin ligases, recruitment of phosphatases, and inhibition of cytokine receptor signaling [52]. Checkpoint inhibitors against this T cell exhaustion marker have been demonstrated to be effective in both preclinical models and patients.

In this study, we found that CXCR5, CXCR6 and CXCL13 involved in the pathway of chemotaxis are over-expressed in the network (Supplementary Table 1, Fig. 4c, f). Study demonstrated that CXCR5 and CXCL13 were also highly expressed in TILs from NSCLC cancer [53]. Go and pathway analysis indicated CXCR6 and CXCR5 were also implicated in the locomotory behavior molecule pathway in our study (Supplementary Table 1 and Fig. 4f). Previous research revealed that systemic used of IFN-γ could drive inflammation and facilitate T cell infiltration in cold tumors [54]. Therefore, we speculate that the above-mentioned highly expressed molecules might contribute to the infiltration of T cells, and consequently results in a hot TME in BC. In addition, JUN, a putative transforming and remodeling gene, high expression of these molecules in CD4^+^ TILs might contribute the cells epigenome vulnerable to immunoediting with the cancer development. Meanwhile, Jak − STAT3 signaling pathway was significantly activated which is well-known for its key role in the recruitment of myeloid-derived suppressor cells to TME and exertion of their immunosuppressive effect [55].

## Conclusion

Our study revealed that cytotoxic and memory T cells significantly decreased, whereas regulatory T cells and apoptotic T cells markedly increased in TNBC patients. Tumors infiltrated with polarized regulatory T cells were suggested an immunosuppressive microenvironment and a poorer prognosis of TNBC patients [56, 57]. By comparing the profiles of the whole immune cells from TNBC tissue with that from normal breast tissue, we found that although Treg cells increased, but there is no significance (Supplementary Fig. 4). The reason for this phenomenon may due to the variety subsets of the lymphocytes in whole immune cells in which the small number of CD4^+^ T Treg lead to its lots of information was concealed by the gene expressive abundance of all lymphocytes. Meanwhile, the expression level of CTLA4 and FAS were markedly increased in intratumoral CD4^+^ T cell, which may play an essential role in maintaining self-tolerance as well as suppressing anticancer immunity. On the other hand, CXCR5, CXCR6 and CXCL13 were involved in chemotaxis, locomotory behavior and leukocyte migration (Supplementary Table 1, Fig. 4c, f). Over-expression of CXCR5, CXCR6 and CXCL13 may be associated with CXCL13/CXCR5 signaling axis involved in immune cell trafficking to the TME. However, this signaling axis could also modulate cancer cell ability to grow, proliferate, invade, and metastases through IL-10 secretion as well as recruit Treg cells and myeloid-derived immunosuppressive cells to the tumor microenvironment [58–60]. Therefore, for generating novel therapeutics, it will be important to develop Treg cell-targeted therapies or facilitate precision medicine focusing on CXCL13/CXCR5 signaling axis to enhance the adaptive antitumor immunity. Nevertheless, it reminds us of some limitations in the analysis since various gene interactions result in different various cellular conditions and functional heterogeneity of the CD4^+^ TILs. This exploratory analysis still provides suggestions of potential candidate genes as well as the signal pathways underlying tumor-infiltrating CD4^+^ T cells in TME and bestows a theranostic perspective to the current trend of research.

### Supplementary Information

Below is the link to the electronic supplementary material.

Supplementary Fig. 1. IHC staining and quantitative evaluation of CD4+ T cells in breast cancer and normal tissues. a IHC staining of CD4+ T cells in normal breast tissue. b Proportion of the CD4+ T cells in normal breast tissue. c Proportion of the CD4+ T cells in stroma of breast tumor (Figure 1a). d Proportion of the CD4+ T cells in stroma and infiltrated among the cancer cells. (n =6, P < 0.05, 200X) (TIFF 2606 KB)

Supplementary Fig. 2. PPI networks associated with the DEGs derived by comparing the expression profile of CD4+ T cells from peripheral blood of TNBC patients with the cells from healthy donor blood. a Network of the DEGs. b Network derived from panel A with first neighbors associated with the core proteins CXCR6 and CXCL13. c Network derived from panel A with first neighbors associated with the core proteins FN1 and IFNG. d Significant hub nodes extracted from networks b and c (TIF 20121 KB)

Supplementary Fig. 3. Quantitative evaluation of CD4+ T cells and apoptotic T cells. a Immunofluorescence labeling of CD4+ T cells infiltrated in normal breast tissue. b Proportion of the CD4+ T cells in normal breast tissue. c Proportion of the CD4+ T cells in tumor tissue (Fig. 6b). d Proportion of the CD4+ T cells in tumor tissue (Fig. 6c). e Proportion of the CD4+ T cells in tumor tissue (Fig. 6d). e Proportion of the apoptotic T cells in breast tumor tissue (Fig. 6e). f Detection of apoptotic T cells by TUNEL in normal breast tissue. g Proportion of the apoptotic T cells in normal breast tissue. (n =6, P < 0.05, 200X) (TIFF 12124 KB)

Supplementary Fig. 4. Expression profiles of the whole immune cells infiltrated in TNBC compared with that from normal breast sample (TIFF 455 KB)

Supplementary file5 (DOCX 14 KB)

## Data Availability

The data of this study have been deposited in the Gene Expression Omnibus repository GSE150814 (https://www.ncbi.nlm.nih.gov/geo/query/acc.cgi?acc=GSE150814).
