# Supplementary figures and images for: Comprehensive genomic and immunophenotypic analysis of CD4 T cell infiltrating human triple-negative breast cancer
Source: Cancer Immunol Immunother. 2020 Dec 10;70(6):1649–65. doi: 10.1007/s00262-020-02807-1 (PMC8139937; doi:10.1007/s00262-020-02807-1)

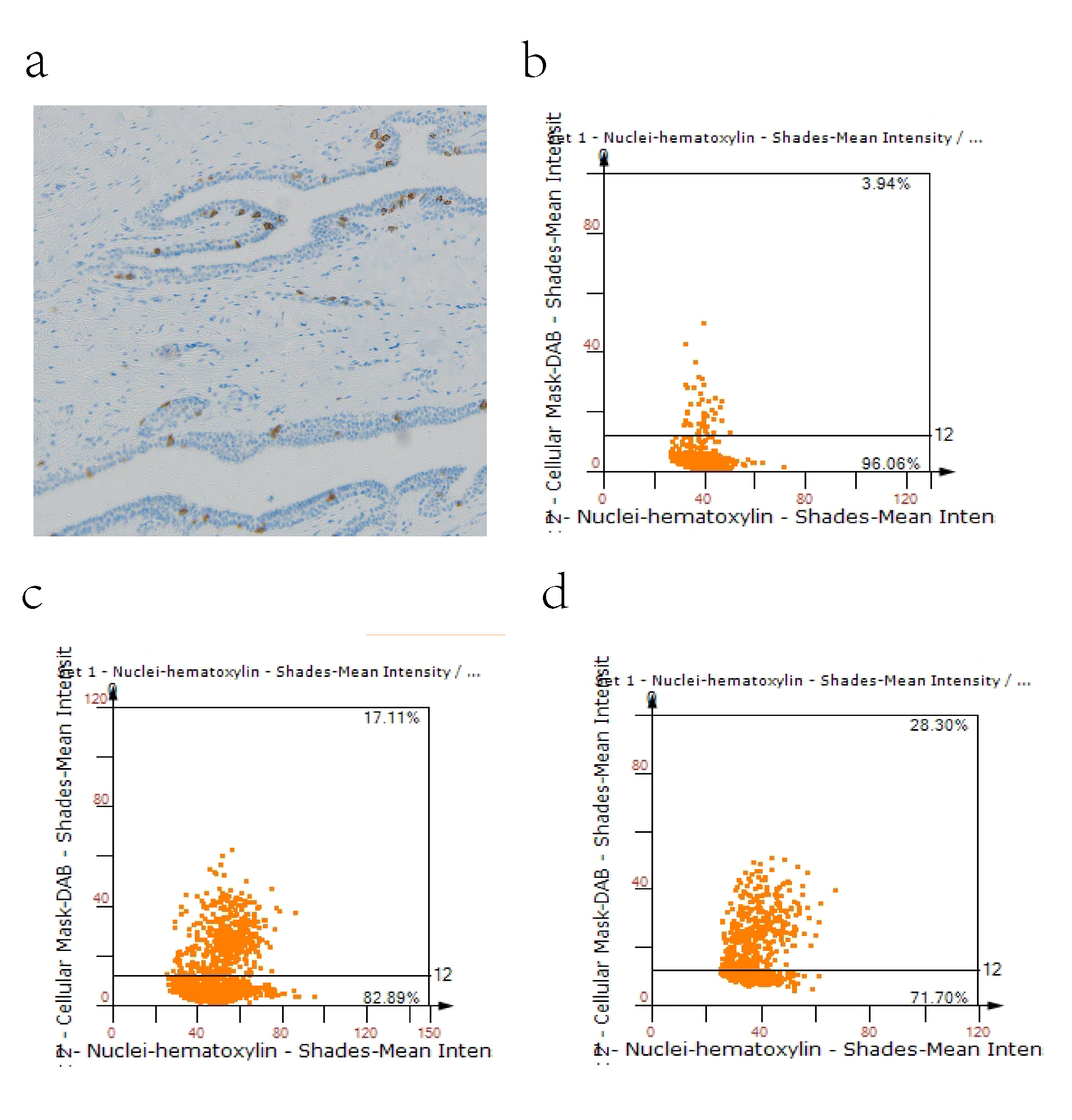

Supplement: Supplementary file 1 — Supplementary Fig. 1. IHC staining and quantitative evaluation of CD4+ T cells in breast cancer and normal tissues. a IHC staining of CD4+ T cells in normal breast tissue. b Proportion of the CD4+ T cells in normal breast tissue. c Proportion of the CD4+ T cells in stroma of breast tumor (Figure 1a). d Proportion of the CD4+ T cells in stroma and infiltrated among the cancer cells. (n =6, P < 0.05, 200X) (TIFF 2606 KB) [file 262_2020_2807_MOESM1_ESM.tiff]

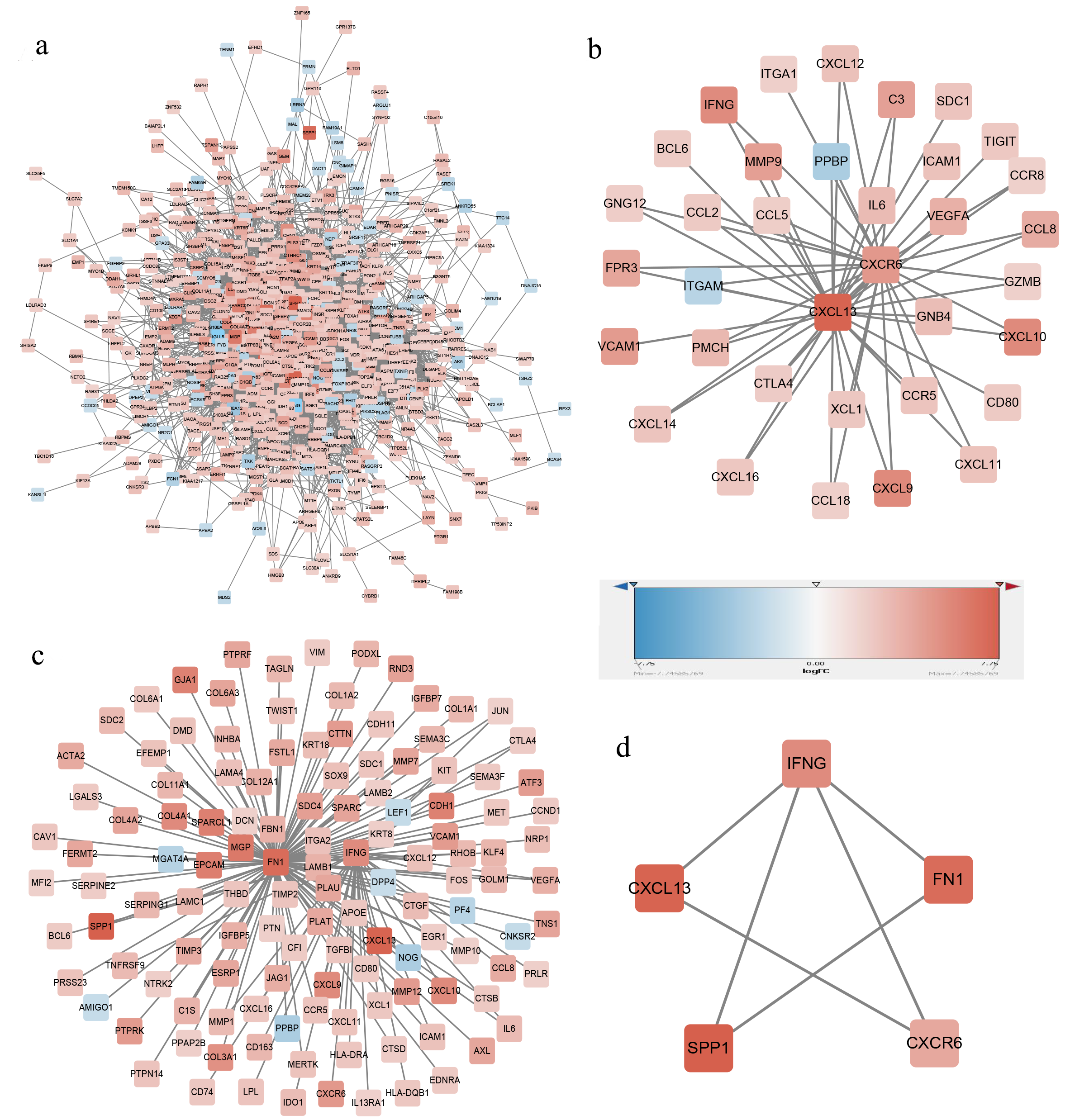

Supplement: Supplementary file 2 — Supplementary Fig. 2. PPI networks associated with the DEGs derived by comparing the expression profile of CD4+ T cells from peripheral blood of TNBC patients with the cells from healthy donor blood. a Network of the DEGs. b Network derived from panel A with first neighbors associated with the core proteins CXCR6 and CXCL13. c Network derived from panel A with first neighbors associated with the core proteins FN1 and IFNG. d Significant hub nodes extracted from networks b and c (TIF 20121 KB) [file 262_2020_2807_MOESM2_ESM.tif]

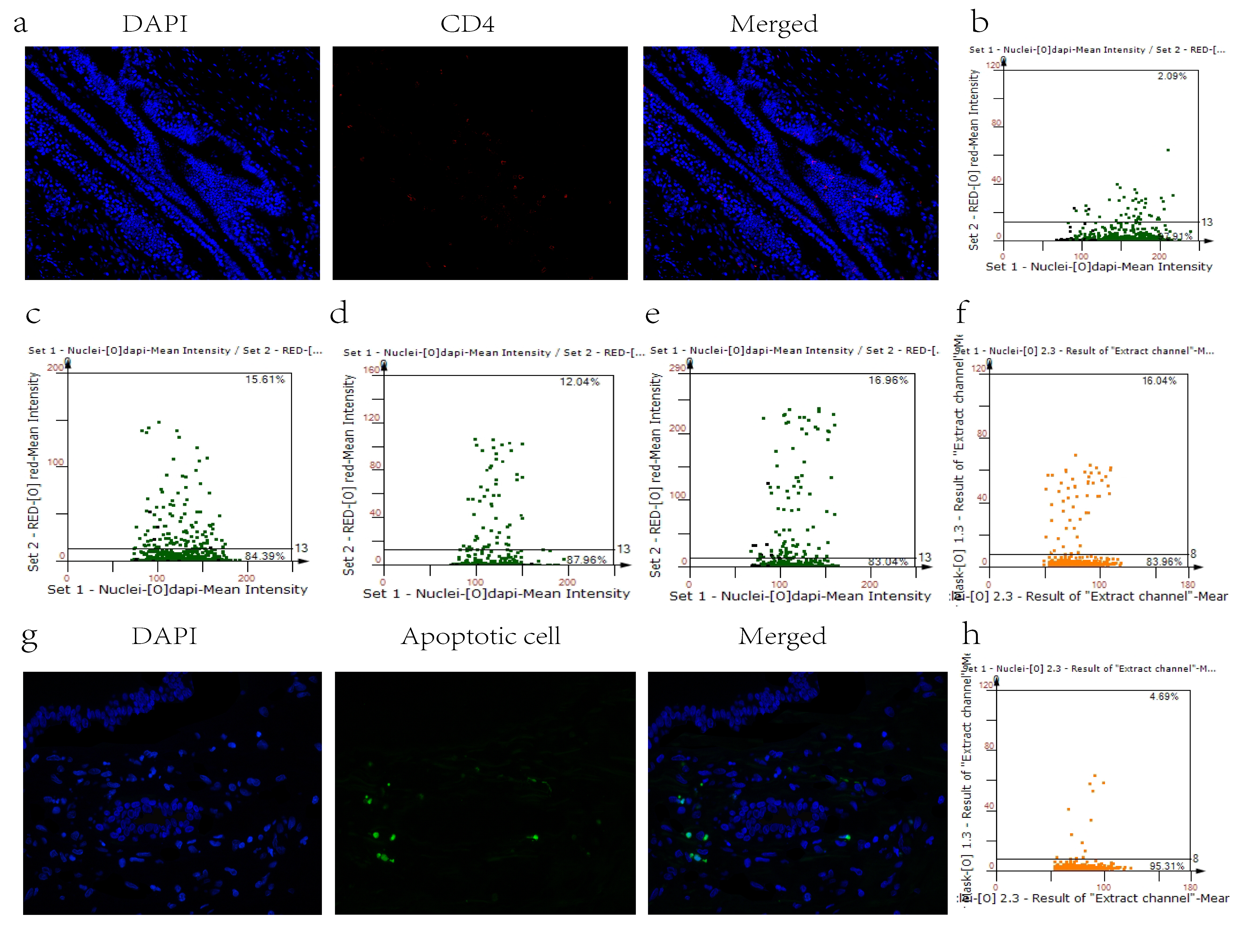

Supplement: Supplementary file 3 — Supplementary Fig. 3. Quantitative evaluation of CD4+ T cells and apoptotic T cells. a Immunofluorescence labeling of CD4+ T cells infiltrated in normal breast tissue. b Proportion of the CD4+ T cells in normal breast tissue. c Proportion of the CD4+ T cells in tumor tissue (Fig. 6b). d Proportion of the CD4+ T cells in tumor tissue (Fig. 6c). e Proportion of the CD4+ T cells in tumor tissue (Fig. 6d). e Proportion of the apoptotic T cells in breast tumor tissue (Fig. 6e). f Detection of apoptotic T cells by TUNEL in normal breast tissue. g Proportion of the apoptotic T cells in normal breast tissue. (n =6, P < 0.05, 200X) (TIFF 12124 KB) [file 262_2020_2807_MOESM3_ESM.tiff]

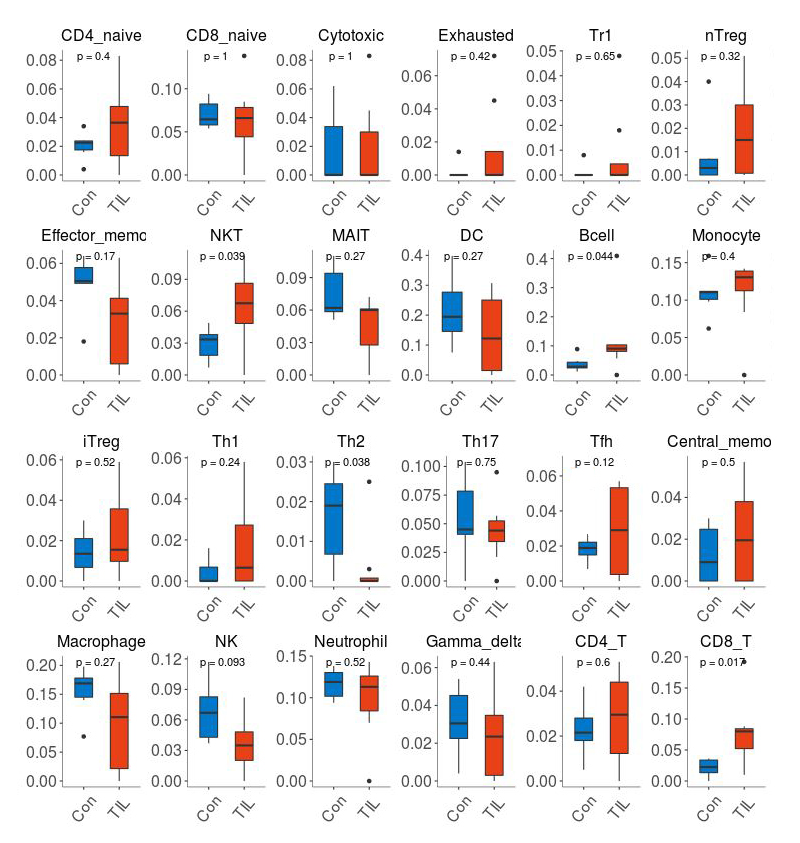

Supplement: Supplementary file 4 — Supplementary Fig. 4. Expression profiles of the whole immune cells infiltrated in TNBC compared with that from normal breast sample (TIFF 455 KB) [file 262_2020_2807_MOESM4_ESM.tiff]
