# Supplementary material for: Comprehensive genomic and immunophenotypic analysis of CD4 T cell infiltrating human triple-negative breast cancer
Source: Cancer Immunol Immunother. 2020 Dec 10;70(6):1649–65. doi: 10.1007/s00262-020-02807-1 (PMC8139937; doi:10.1007/s00262-020-02807-1)
Supplement: Supplementary file 5 — Supplementary file5 (DOCX 14 KB) [file 262_2020_2807_MOESM5_ESM.docx]

Supplementary Table 1. Enrichment analysis of the DEGs from network C.

| ID | Terms | | | Count | | PValue | | Genes |
| --- | --- | --- | --- | --- | --- | --- | --- | --- |
| GO:0006935 | chemotaxis | | 14 | | 9.71E-17 | | | CXCR5, CXCL13, CXCR6，CXCL2, FPR1, CCL5, C5AR1, CXCL3, CCR1, ITGAM, PPBP, CCL20, CX3CR1, IFNG |
| GO:0042330 | taxis | | 14 | | 9.71E-17 | | | CXCL13, CXCL2, CXCR5，FPR1, CCL5, ITGAM, PPBP, CCL20, C5AR1, CCR1, CX3CR1, CXCL3,IFNG, IL1B, XCL1 |
| GO:0007626 | locomotory behavior | | 14 | | 1.11E-13 | | | CXCL13, CCL5, CXCR6， C5AR1, CXCR5, CCR1, CXCL3, CXCL2, FPR1,ITGAM, PPBP, CCL20, CX3CR1, IFNG |
| GO:0006955 | immune response | | 18 | | 3.05E-13 | | | CR1, C5AR1, CCR1, CXCL3, GPSM3, CXCL2, CTLA4, EOMES, TLR2, CCL5, PDCD1, TNFSF13B, PPBP, CCL20, CXCL13, IFNG, IL1B, XCL1 |
| GO:0007166 | cell surface receptor linked signal transduction | | 23 | | 5.12E-11 | | | ADAM10, C5AR1, PMCH, CCR1, CXCL3, CXCL2, GABBR1, FPR1, TLR2, ANXA1, CCL5, ITGAM, STAT3, RGS13, P2RY13, S1PR1, PPBP, CXCR5, CCL20, CXCR6, CX3CR1, IFNG, GNG4 |
| GO:0006952 | defense response | 15 | | | 2.09E-10 | | | CR1, C5AR1, CCR1, CXCL3, CXCL2, TLR2, ANXA1, CCL5, STAT3, PPBP, CCL20, CXCL13, CX3CR1, IFNG, IL1B |
| GO:0007186 | G-protein coupled receptor protein signaling pathway | 17 | | | 6.79E-09 | | | C5AR1, PMCH, CCR1, CXCL3, CXCL2, GABBR1, FPR1, CCL5, RGS13, P2RY13, S1PR1, PPBP, CXCR5, CCL20, CXCR6, CX3CR1, GNG4 |
| GO:0006954 | inflammatory response | 11 | | | 8.15E-09 | | | CR1, CCL20, CXCL13, CXCL3, CCR1, CXCL2, ANXA1, TLR2, IL1B, CCL5, STAT3 |
| GO:0048584 | positive regulation of response to stimulus | 9 | | | 1.51E-07 | | | CR1, ADAM10, S1PR1, TNFSF13B, PMCH, TLR2, IL1B, IL21, CCL5 |
| GO:0050900 | leukocyte migration | 8 | | | 3.79E-07 | | | VCAM1, CXCR6，CXCL13, IFNG, CXCR5，IL1B, CCL5, ITGAM |
|  |  | | |  |  | |  | |
